# Near-Optimal Graph Signal Sampling by Pareto Optimization

**DOI:** 10.3390/s21041415

**Published:** 2021-02-18

**Authors:** Dongqi Luo, Binqiang Si, Saite Zhang, Fan Yu, Jihong Zhu

**Affiliations:** 1 Department of Computer Science and Technology, Tsinghua University, Beijing 100084, China; ldq17@mails.tsinghua.edu.cn (D.L.); zhang-st17@mails.tsinghua.edu.cn (S.Z.); yuf18@mails.tsinghua.edu.cn (F.Y.); 2 School of Instrumentation Science and Opto-Electronics Engineering, Beijing Information Science and Technology University, Beijing 100192, China; sibinqiang09@tsinghua.org.cn; 3 Department of Precision Instrument, Tsinghua University, Beijing 100084, China

**Keywords:** graph signal processing, graph signal sampling, supermodularity ratio, evolutionary algorithms, pareto optimization

## Abstract

In this paper, we focus on the bandlimited graph signal sampling problem. To sample graph signals, we need to find small-sized subset of nodes with the minimal optimal reconstruction error. We formulate this problem as a subset selection problem, and propose an efficient Pareto Optimization for Graph Signal Sampling (POGSS) algorithm. Since the evaluation of the objective function is very time-consuming, a novel acceleration algorithm is proposed in this paper as well, which accelerates the evaluation of any solution. Theoretical analysis shows that POGSS finds the desired solution in quadratic time while guaranteeing nearly the best known approximation bound. Empirical studies on both Erdos-Renyi graphs and Gaussian graphs demonstrate that our method outperforms the state-of-the-art greedy algorithms.

## 1. Introduction

**Figure 1:**
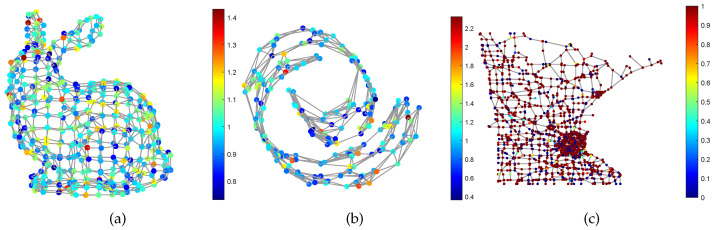
Illustration of smooth graph signals. The signals are supported on (**a**) The stanford bunny graph; (**b**) The swiss roll graph; (**c**) The Minnesota road graph.

Graph Signal Processing (GSP) is a powerful tool for irregular data modeling and processing, and has attracted extensive attention from the signal processing [1,2], machine learning [3], and computer vision communities [4,5]. Traditional signal processing usually treat the data as a sequence of vectors, then analyze the features in the time domain or frequency domain, and try to extract information from the features. This implies that the data rely on an Euclidean space $R^{n}$. However, in many real-world applications such as computer graphics [6] and machine learning [7], this assumption is not accurate enough. For example, the data captured by sensors in a sensor network are always interconnected to each other, and they may rely on a manifold embedded in an Euclidean space. Moreover, a RGB image lies within the pixel space $R^{H\times W\times 3}$, but the dimension of the natural image space is usually much lower. GSP brings the signal from the time (spatial) domain to the node domain, and allows us to use a similar tool in traditional signal processing, such as Fourier transform and wavelet analysis, to analyze the graph signals, and then extract the latent information. As an illustration, Figure 1 shows the graph signals supported on the Stanford bunny graph, the Swiss roll graph and the Minnesota road graph respectively.

However, in some applications, the underlying graph is very large, which makes it very expensive to collect and process the data from all nodes. Graph signal sampling is a basic problem in GSP that aims to select a subset with the minimal reconstruction error from the graph node set. Hence, we just need to collect the signal from a small-sized sampling set, and the complete graph signal can be obtained via optimal graph signal reconstruction. Graph signal sampling has been widely used in many fields, e.g., sensor placement [8] and 3D point-cloud sampling [9]. Previous works [10,11] first considered the graph signal reconstruction problem, which aims to recover the whole graph signal from partial observations, then formulated the graph signal sampling problem as a subset of nodes with the minimal optimal reconstruction error. This sampling problem can be regarded as a subset selection problem, which plays a fundamental role in many fields, such as sparse reciprocal graphical model estimation [12] and sparse plus low rank graphical model estimation [13]. This problem is proved to be generally NP-hard [14], so we may not find a global optimal solution of this problem in polynomial time. A sub-optimal method is the greedy algorithm, which selects an element with the greatest improvement to the objective function at each iteration. The greedy algorithm is very simple, but also very effective. In [15], it is proved that the greedy algorithm achieves $1-e^{-\gamma }$ approximation ratio for normalized (that means f(⌀)=0,f(V)=1) monotonic weak submodular objective functions in general subset selection problems, where γ is the submodularity ratio of the objective function. For submodular objective functions we have γ≥1, then the greedy algorithm guarantees 1−1/e performance, which is the best-so-far theoretical guarantee.

Recently, many Multi-Objective Evolutionary Algorithms (MOEAs) such as the Pareto Optimization for Subset Selection (POSS) algorithm have exhibited better performance than greedy algorithms do [16,17,18]. POSS treats the cardinality constraint as an additional objective function of the problem, then the original problem is transformed to a Bi-objective Optimization Problem (BOP). POSS adopts the evolutionary mutation operation to search for different Pareto optimal solutions of the BOP, and finally select a desired Pareto optimal solution.

Though POSS has been proved to be a powerful tool to solve the subset selection problem, it is infeasible to directly apply it to the graph signal sampling problem, since the evaluation of the optimal reconstruction error is very expensive. In this work, we propose the Pareto Optimization for Graph Signal Sampling (POGSS) based on POSS, and an acceleration algorithm is proposed. By introducing the supermodularity ratio of the objective function, we prove that this method theoretically achieves comparable performance as greedy algorithms do in polynomial time, and show that the supermodularity ratio is bounded as well. Our major contributions can be summarized as follows:

1. Inspired by POSS, we formulate the graph signal sampling problem as a subset selection problem, and solve this problem by employing Pareto optimization, which has been proved to be superior to greedy methods.
2. To accelerate the objective estimation procedure, we propose a novel acceleration algorithm based on matrix inversion lemma. Our acceleration algorithm is scalable since it can be used to accelerate the evaluation of any solution, thus that can be directly applied to other evolutionary-based methods (e.g., POSS with recombination [17]).
3. We provide comprehensive theoretical analyses of the proposed algorithm, including complexity analysis, Pareto optimality analysis and error bound analysis.

The remainder of this paper is organized as follows. We present the preliminaries and formulate the problem in Section 2.1. We give a brief introduction to the POSS method and introduce the Pareto optimization for graph signal sampling (POGSS) algorithm in Section 3. In Section 4, we provide comprehensive theoretical analyses of POGSS. The Numerical results and the conclusions are presented in Section 5 and Section 6, respectively.

## 2. Preliminaries

### 2.1. Notations

In this paper, we use the bold uppercase letters (e.g., A) to denote matrices and use bold lowercase letters (e.g., a) to denote vectors. (i,j)-th entry of A is denoted as $A_{ij}$ or ${\left[A\right]}_{ij}$. ${\left(\cdot \right)}^{T}$ denote the transpose, ${\left(\cdot \right)}^{-1}$ denote the inverse, ${\left(\cdot \right)}^{†}$ denote the Moore-Penrose pseudo-inverse. Tr(·) denote the trace. E[·] denote the expectation. diag(a) stands for a diagonal matrix with the diagonal entries given by a vector a. Sets are denoted by calligraphic letters, e.g., A (except the set of real number, which is denoted as R). [n] denote the set of all integers from 1 to *n*, i.e., [n]={1,2,⋯,n}. ⌈·⌉ denote the ceil operator, e.g., ⌈0.1⌉=1.

### 2.2. Graph Signal Processing

We consider a weighted, undirected graph G={V,E}, where V is the set of nodes with |V|=n, E is the set of edges. Let $A\in R^{n\times n}$ be the adjacency matrix of G, where $A_{ij}$ is the weight of the edge between nodes *i* and *j*. The graph signal x is a function that maps each node in V to a real number, and can be written as a vector with its’ *i*-th entry denotes the signal value at nodes *i*.

Defined the unnormalized graph Laplacian matrix of G as

$$\begin{array}{c} L=D-A, \end{array}$$

where D is a diagonal degree matrix with $D_{ii}=\sum_{j=1}^{n}A_{ij}$. Then we write the eigenvalue decomposition of the graph Laplacian matrix as

$$\begin{array}{c} L=V\Lambda V^{T}, \end{array}$$

where $\Lambda =\{0=\lambda_{1},\lambda_{2},\cdots ,\lambda_{n}\}$ is a diagonal matrix of graph frequencies, and V is the matrix of graph Fourier bases. Then the graph Fourier transform of x is defined as $\bar{x}=V^{T}x$, and the inverse Fourier transform is given by $x=V\bar{x}$. We say a graph signal x is K-bandlimited, if its’ graph Fourier coefficients indexed by V\K are zero, i.e., ${\bar{x}}_{V\backslash K}=0$. In [19], it was proved that if the sampling set size is not smaller than |K|, then the K-bandlimited graph signal can be perfectly recovered. However, in many applications we can only observe noise corrupted signals, we cannot find a perfect estimator under noise. Consequently, Bayesian estimation is adopted in the graph signal reconstruction problem.

### 2.3. Problem Formulation

Let x be the original noise-free graph signal, and n be the noise vector. In the *graph signal reconstruction* problem, only the noise corrupted samples $y_{S}$ in a sampling set S⊆V are observable, thus we take the observation model as

(1) $$\begin{array}{c} \left\{\begin{array}{cc} \; & y=x+n,\\ \; & y_{S}=C_{S}y, \end{array}\right. \end{array}$$

where $C_{S}\in {\left\{0,1\right\}}^{|S|\times n}$ is the sampling matrix. We assume that n is a zero-mean vector with covariance matrix $\sigma^{2}I$, x is K-bandlimited and ${\bar{x}}_{K}$ is a zero-mean vector with covariance matrix $\bar{\Sigma }$, hence the covariance matrix of x is $\Sigma =V_{K}\bar{\Sigma }V_{K}^{T}$. Graph signal reconstruction is to approximately recover the original graph signal x from partial noisy observation $y_{S}$ via a linear transform $\Phi_{S}\in R^{n\times |S|}$, i.e.,

(2) $$\begin{array}{c} \hat{x}\left(\Phi_{S}\right)=\Phi_{S}y_{S}. \end{array}$$

The error covariance matrix with respect to $\Phi_{S}$ is defined as

$$\begin{array}{c} K_{S}\left(\Phi_{S}\right)=E\left[\left(x-\hat{x}\left(\Phi_{S}\right)\right){\left(x-\hat{x}\left(\Phi_{S}\right)\right)}^{T}\right]. \end{array}$$

To find the optimal reconstruction matrix $\Phi_{S}^{★}$, one needs to minimize the scalar loss function $J\left(\Phi_{S}\right)=z^{T}\Phi_{S}z$ for all $z\in R^{n}$. The derived optimal recover operator is [10]

(3) $$\begin{array}{c} \Phi_{S}^{★}=\Sigma C^{T}{\left(C\left(\Sigma +\sigma^{2}I\right)C^{T}\right)}^{†}, \end{array}$$

and the correspongding error covariance matrix is given by

(4) $$\begin{array}{c} K_{S}^{★}=K_{S}\left(\Phi_{S}^{★}\right)=V_{K}{\left({\bar{\Sigma }}^{-1}+\sigma^{-2}\sum_{i\in S}v_{i}v_{i}^{T}\right)}^{-1}V_{K}^{T}, \end{array}$$

where $V_{K}^{T}=\left[v_{1},\cdots ,v_{n}\right]$, and the optimal mean squared error (MSE) is given by

(5) $$\begin{array}{c} {\mathrm{MSE}}_{S}^{★}=E\left[∥x-\hat{x}\left(\Phi_{S}^{★}\right){∥}_{2}^{2}\right]=\mathrm{Tr}\left(K_{S}^{★}\right). \end{array}$$

The goal of *graph signal sampling* is to select a subset S⊆V with at most *k* nodes, which minimizes the optimal reconstruction error ${\mathrm{MSE}}_{S}^{★}$, i.e., finding

(6) $$\begin{array}{c} \begin{array}{cc} S^{★} & ={\arg\min}_{S\subseteq V,|S|\leq k}\;{\mathrm{MSE}}_{S}^{★}\\ \; & ={\arg\min}_{S\subseteq V,|S|\leq k}\;\mathrm{Tr}\left(K_{S}^{★}\right). \end{array} \end{array}$$

Define ${\bar{K}}_{S}={\left({\bar{\Sigma }}^{-1}+\sigma^{-2}\sum_{i\in S}v_{i}v_{i}^{T}\right)}^{-1}$, $M=V_{K}^{T}V_{K}$. By using the circular commutation property of trace, (6) is simplified to

(7) $$\begin{array}{c} S^{★}={\arg\min}_{S\subseteq V,|S|\leq k}\;\mathrm{Tr}\left(M{\bar{K}}_{S}\right) \end{array}$$

## 3. Method

In this study, we propose to solve (7) by Pareto optimization. For representable convenience, we use the binary vector $s\in {\left\{0,1\right\}}^{n}$ to represent the subset S⊆V, the entry $s_{i}=1$ means that the node $v_{i}$ is included in S and $s_{i}=0$ otherwise. We do not distinguish between the two notations. Please note that the original problem is a constrained problem with single objective function, we now reformulate this problem as an unconstrained BOP:

(8) $$\begin{array}{c} {\arg\min}_{s\in {\left\{0,1\right\}}^{n}}\;f\left(s\right)={\left(f(s),\;c(s)\right)}^{T}, \end{array}$$

where

$$\begin{array}{cc} \; & f\left(s\right)={\mathrm{MSE}}_{S}^{★}=\mathrm{Tr}\left(M{\bar{K}}_{S}\right),\\ \; & c\left(s\right)=\left\{\begin{array}{cc} {∥s∥}_{0}, & {∥s∥}_{0}<2k\\ +\infty , & {∥s∥}_{0}\geq 2k\;\mathrm{or}\;{∥s∥}_{0}=0 \end{array}\right.. \end{array}$$

To compare two solutions, the Pareto dominance relationship and the Pareto optimality are defined as follows:

**Definition** **1** (Pareto dominance). *Let $s_{1}$ and $s_{2}$ be two solutions, then*

- (Weak dominance) $s_{1}$ weakly dominates $s_{2}$ ($s_{1}⪯s_{2}$), if and only if $f\left(s_{1}\right)\leq f\left(s_{2}\right)$*and*$c\left(s_{1}\right)\leq c\left(s_{2}\right)$;
- (Dominance) $s_{1}$ dominates $s_{2}$ ($s_{1}≺s_{2}$), if and only if $s_{1}⪯s_{2}$*and*$\left(f\left(s_{1}\right)<f\left(s_{2}\right)\right.$*or*$\left.c\left(s_{1}\right)<c\left(s_{2}\right)\right)$.

**Definition** **2** (Pareto optimality). *We say $s^{★}$ is a Pareto optimal solution with respect to M, if there is no solution s∈M such that $s≺s^{★}$.*

To summarize, a solution $s_{1}$ is said to weakly dominates $s_{2}$ if $s_{1}$ performs not worse than $s_{2}$ for all metrics, and for two solutions such that $s_{1}⪯s_{2}$, we say $s_{1}$ dominates $s_{2}$ if there is at least one metric for which $s_{1}$ strictlly outperforms $s_{2}$. $s^{★}$ is said to be a Pareto optimal solution with respect to M if there is no solution in M that dominates $s^{★}$.

### 3.1. Pareto Optimization for Subset Selection (Poss)

POSS is an evolutionary algorithm for subset selection, and its overall procedure is shown in Algorithm 1. Denote $T=2^{V}$ as the set of all solutions, M⊆T as the set of all solutions found so far. POSS maintains a set P⊆M of all Pareto optimal solutions with respect to M. At each step, POSS first randomly selects a solution from the set of Pareto optimal solutions P, and then adopts the mutation operator to search for a potential Pareto optimal solution. If there is no solution that dominates the generated solution $s^{\prime }$, then POSS includes the generated solution to P, and remove the solutions which are weakly dominated by $s^{\prime }$ from P; Otherwise POSS discards the solution s. Such update strategy in POSS guarantees that if P is the Pareto optimal solution set at current iteration, then the updating operation does not change the optimality of P. After *T* iterations, POSS outputs a solution $s^{★}\in P$ with the minimal reconstructed error and a cardinality smaller than *k*, i.e., $s^{★}=\arg\min_{s\in P,{∥s∥}_{0}\leq k}f\left(s\right)$.

**Algorithm 1** POSS**Input:**  An universal set V, $f:2^{V}\to R$, k∈[n], $T\in N_{+}$.**Output:**  a subset S⊆V with at most *k* nodes.  1:  $s={\left\{0\right\}}^{n}$, P={s}.  2:  **for**
t=1,2,⋯,T
**do**  3:    Select s from P uniformly at random.  4:    Generate $s^{\prime }$ by bit-wise flipping with probability $\frac{1}{n}$.  5:    **if**
∄z∈P such that $z≺s^{\prime }$
**then**  6:      $P=\left(P\backslash \left\{z\in P|s^{\prime }⪯z\right\}\right)\cup \left\{s^{\prime }\right\}$.  7:    **end if**  8:  **end for**  9:  $s^{★}={\arg\min}_{s\in P,{∥s∥}_{0}\leq k}f\left(s\right)$.

### 3.2. Proposed Pareto Optimiazation for Graph Signal Sampling (Pogss)

However, due to the general $O(n^{3})$ complexity of the matrix inversion operation, the evaluation step of f(s) is very expensive in general, which means we cannot efficiently compare two solutions. Please note that at each iteration the new solution is generated from a solution evaluated before, we can accelerate the evaluation of the objective function by leveraging the information of the previous solution. More explicitly, by using the matrix inversion lemma [20] we deduced the following lemma:

**Lemma** **1.** *Let S⊆V, then for all $v_{1}\in V\backslash S,v_{2}\in S$, we have*

(9) $$\begin{array}{cc} \; & {\bar{K}}_{S\cup \{v_{1}\}}={\bar{K}}_{S}-\frac{{\bar{K}}_{S}v_{v_{1}}v_{v_{1}}^{T}{\bar{K}}_{S}}{\sigma^{2}+v_{v_{1}}^{T}{\bar{K}}_{S}v_{v_{1}}} \end{array}$$

(10) $$\begin{array}{cc} \; & {\bar{K}}_{S\backslash \{v_{2}\}}={\bar{K}}_{S}+\frac{{\bar{K}}_{S}v_{v_{2}}v_{v_{2}}^{T}{\bar{K}}_{S}}{\sigma^{2}-v_{v_{2}}^{T}{\bar{K}}_{S}v_{v_{2}}} \end{array}$$

Let $t_{I}=s^{\prime }\backslash s=\left\{t_{I_{1}},\ldots ,t_{I_{l}}\right\}$ be the elements to be included at the current iteration, $t_{E}=s\backslash s^{\prime }=\left\{t_{E_{1}},\ldots ,t_{E_{m}}\right\}$ denote the elements to be excluded, and $t_{R}=s\cap s^{\prime }$ denote the elements that will be retained. Instead of directly computing the ${\bar{K}}_{s^{\prime }}$, we propose to compute ${\bar{K}}_{s\cup t_{I}}$ firstly, and then compute the desired ${\bar{K}}_{s^{\prime }}={\bar{K}}_{\left(s\cup t_{I}\right)\backslash t_{E}}$. By using Lemma 1, we can easily compute ${\bar{K}}_{s\cup t_{I}}$ by including the elements in $t_{I}$ to s one by one, and then compute ${\bar{K}}_{s^{\prime }}$ by excluding the elements in $t_{E}$ from $s\cup t_{I}$ separately. The full procedure of POGSS is described in Algorithm 2, and the solution flipping algorithm for acceleration is presented in Algorithm 3.

Denote $N_{t}=|t_{I}\left|+\right|t_{E}|$ as the number of bits that will be flipped at iteration *t*, then we have $E(N_{t})=1$, which reveals the fact that we are expected to flip only 1 bit at each iteration. Please note that the evaluation of (9) and (10) can be done at a cost of $O(n^{2})$, so the cost of evaluating the objective function in Algorithm 2 is expected to be $O(n^{2})$, while the naive matrix inversion update requires a cost of $O(n^{3})$. Therefore, this proposed procedure significantly accelerates the algorithm.

**Algorithm 2** POGSS**Input:**  Σ, V, integer k∈[n], $T\in N_{+}$.**Output:**  a subset $S^{★}\subseteq V$ with at most *k* nodes.  1:  $s={\left\{0\right\}}^{n}$, ${\bar{K}}_{s}=\Sigma$, P={s}.  2:  **for**
t=1,2,⋯,T
**do**  3:    Select s from P uniformly at random.  4:    Generate $s^{\prime }$ by bit-wise flipping with probability $\frac{1}{n}$.  5:    $\mathrm{Compute}\;{\bar{K}}_{s^{\prime }}\;\mathrm{by}\;\mathrm{invoking}\;\mathrm{Algorithm}\;,\;\mathrm{then}\;\mathrm{evaluate}\;\mathrm{the}\;\mathrm{objective}\;\mathrm{function}f\left(s^{\prime }\right)=\mathrm{Tr}\left(M{\bar{K}}_{s^{\prime }}\right).$  6:    **if**
∄z∈P such that $z≺s^{\prime }$
**then**  7:      $P=\left(P\backslash \left\{z\in P|s^{\prime }⪯z\right\}\right)\cup \left\{s^{\prime }\right\}$.  8:    **end if**  9:  **end for**10:  Output $s^{★}={\arg\min}_{s\in P,{∥s∥}_{0}\leq k}f\left(s\right)$.

**Algorithm 3** Solution Flipping**Input:**  $s,{\bar{K}}_{s},s^{\prime }$.**Output:**  ${\bar{K}}_{s^{\prime }}$.  1:  Initialize $s_{0}=s$, ${\bar{K}}_{s_{0}}={\bar{K}}_{s}$.  2:  Compute the difference set $t_{I}=s^{\prime }\backslash =\left\{t_{I_{1}},\cdots ,t_{I_{l}}\right\}$ and $t_{E}=s\backslash s^{\prime }=\left\{t_{E_{1}},\cdots ,t_{E_{m}}\right\}$.  3:  **for**
i=1⋯,l
**do**  4:    $s_{i}=s_{i-1}\cup \left\{t_{I_{i}}\right\}$.  5:    Compute ${\bar{K}}_{s_{i}}={\bar{K}}_{s_{i-1}\cup \left\{t_{I_{i}}\right\}}$ by using (9).  6:  **end for**  7:  **for**
i=1⋯,m
**do**  8:    $s_{l+i}=s_{l+i-1}\backslash \left\{t_{E_{i}}\right\}$.  9:    Compute ${\bar{K}}_{s_{l+i}}={\bar{K}}_{s_{l+i-1}\backslash \left\{t_{E_{i}}\right\}}$ by using (10).10:  **end for**11:  Output ${\bar{K}}_{s^{\prime }}={\bar{K}}_{s_{l+m}}$.

## 4. Theoretical Analysis

In this section, we theoretically analyze the performance of POGSS, including the Pareto optimality of P and the overall reconstruction error of POGSS.

We first show in Proposition 1 that the subset P is the set of *all Pareto optimal solutions* with respect to M.

**Proposition** **1** (Pareto optimality of P). *At any iteration of the algorithm 2, let M be the set of all solutions found so far, then*

- for all s∈P, there is no $s^{\prime }\in M$ such that $s^{\prime }≺s$,
- for all s∈(M\P), there is a solution $s^{\prime }\in M$ such that $s^{\prime }≺s$.

We then analyze the performance of POGSS by introducing the supermodularity ratio. A set function $f:2^{V}\to R_{+}$ is said to be a *supermodular* function, if f(A∪{u})−f(A)≤f(B∪{u})−f(B) holds for any A⊆B⊆V and v∈V\B. For a general criterion *f*, Definition 3 defines how close it is to a supermodular function, i.e., the supermodularity ratio $\gamma_{S,k}\left(f\right)$ [21]. It is easy to prove that *f* is supermodular if and only if $\gamma_{S,k}\left(f\right)\geq 1$ holds for any S and *k*.

**Definition** **3** (Supermodularity ratio). *Let f be a non-negative set function. The submodularity ratio of f with respect to a set S and a parameter k≥1 is*

(11) $$\begin{array}{c} \gamma_{S,k}\left(f\right)=\min_{L\subseteq S,U:|U|\leq k,L\cap U=⌀}\frac{f(L\cup U)-f(L)}{\sum_{u\in U}\left(f\left(L\cup \left\{u\right\}\right)-f\left(L\right)\right)} \end{array}$$

The most time-consuming operation in POGSS is the evaluation of the objective function after bit flipping. So here comes a question: how many bit flipping operations we need to obtain a desired solution? We provide an approximation upper bound of the optimal reconstruction MSE with $O(k^{2}n)$ bits flipped in Theorem 1. Please note that the greedy algorithm guarantees $\left(1-e^{-\gamma_{s^{G},k}}\right)\cdot OPT+e^{-\gamma_{s^{G},k}}f\left(0\right)$ performance, where $s^{G}$ is the solution given by the greedy algorithm, POGSS achieves nearly the best known approximation guarantee as greedy algorithms do in quadratic time.

**Theorem** **1.** *Define $\gamma_{\min}=\min_{{s:∥s∥}_{0}=k-1}\gamma_{s,k}$, let N be the total number of flipped bits, OPT be the optimal value of (7). Then POGSS finds a solution s such that*

$$\begin{array}{c} f\left(s\right)\leq \left(1-e^{-\gamma_{\min}}\right)\cdot OPT+e^{-\gamma_{\min}}f\left(0\right), \end{array}$$

*with*

$$\begin{array}{c} E\left(N\right)\leq 2k^{2}\exp\left(-\frac{n}{n-0.5}\right)n\leq 2ek^{2}n. \end{array}$$

The proof of Theorem 1 relies on Lemma 2 and 3 stated bellow. The complete proof is similar to the proof of theorem 14.1 in [22], which is omitted due to the limited space.

**Lemma** **2.** *For any n≥2,*

$$\begin{array}{c} {\left(1-\frac{1}{n}\right)}^{n-1}\geq e^{-(n-0.5)/n}\geq e^{-1} \end{array}$$

*holds.*

**Lemma** **3** ([16]). *$\forall s\in {\left\{0,1\right\}}^{n}$, there exsits one nodes v∈s such that*

$$\begin{array}{c} f\left(s\right)-f\left(s\cup \left\{v\right\}\right)\geq \frac{\gamma_{s,b}}{b}\left(f(s)-OPT\right) \end{array}$$

Please note that the approximation upper bound in Theorem 1 involves $\gamma_{\min}$, we show in Theorem 2 that $\gamma_{\min}$ is also bounded.

**Proposition** **2** (Lower bound for $\gamma_{\min}$). *The supermodularity ratio satisfies*

$$\begin{array}{c} \gamma_{\min}\geq \frac{1}{k}. \end{array}$$

This conclusion is natural and obvious, since the equality holds when $f\left(L\right)-f\left(L\cup U\right)=\max_{u\in U}f\left(L\right)-f\left(L\cup \left\{u\right\}\right)$, and is also relatively loose. However, it is still meaningful because it is independent of the graph structure and the Signal-to-Noise Ratio (SNR), so it is tighter than the bound in [10] in high SNR scenarios.

## 5. Experiments

In this section, we conduct several experiments to evaluate the performance of POGSS. We first state the experimental settings, and then report the experimental results.

### 5.1. Experimental Settings

Two types of undirected graphs are adopted for evaluation:

- The Erdos-Renyi (ER) graph: For ER graphs, any two nodes are connected with probability *p*, i.e., $P(W_{i,j}=W_{j,i}=1)=p$, $P(W_{i,j}=W_{j,i}=0)=1-p$.
- The Gaussian graph: For Gaussian graphs, we uniformly sample *n* points ${\left\{p_{i}\right\}}_{i=1}^{n}$ in a unit square ${\left[0,1\right]}^{2}$. The edges are first connected with weights
  $$\begin{array}{c} W_{i,j}=W_{j,i}=\exp\left(-\frac{{∥p_{i}-p_{j}∥}_{2}^{2}}{2s^{2}}\right), \end{array}$$
  and we then set the weights which are smaller to some threshold $W_{T}$ to 0.

In our experiments, the number of nodes |V|=n is set to 100 for all graphs, and we set p=0.2 for ER graphs, set $s=0.3,W_{T}=0.6$ for Gaussian graphs.

The graph signal x is sampled from a Gaussian distribution:

(12) $$\begin{array}{c} x∼N(0,V_{K}\bar{\Sigma }V_{K}^{T}) \end{array}$$

where $V_{K}$ is the matrix of graph Fourier bases, $\bar{\Sigma }$ is the covariance matrix of ${\bar{x}}_{K}$. In our experiments, $\bar{\Sigma }$ is generated by $\bar{\Sigma }=PP^{T}$, where $P\in R^{\left|K|\times |K\right|}$ is a random matrix with $P_{i,j}∼N\left(0,1\right)$. The additive Gaussian noise n is adopted in our experiments, and the noise power $\sigma^{2}$ is set to 0.01.

We compare our methods with several algorithms, including the state-of-the-art greedy algorithm, the randomized greedy algorithm, and the convex relaxation algorithm. In all experiments, the number of iterations of POGSS is set to $\left\lceil 2ek^{2}n\right\rceil$ according to Theorem 1. The naive greedy graph siganl sampling algorithm [10] simply selects a node with the largest marginal gain at each iteartion. Assume subset of nodes obtained by the greedy algorithm at the current iteration is S, then the node $v=\arg\max_{v\in V\backslash S}f\left(S\cup \left\{v\right\}\right)$ is included to S. The randomized greedy algorithm [11] uses the similar strategy to the greedy algorithm, but accelerates the algorithm by selecting the best node from a random subset. More explicitly, it first samples a subset R⊂V\S with size −n/klog(ϵ) uniformly, where 0<ϵ<1 is a hyper-parameter, then includes the node $v=\arg\max_{v\in R}f\left(S\cup \left\{v\right\}\right)$ to S. Recall the original problem of graph signal sampling (7)

(13) $$\begin{array}{c} s^{★}=\arg\min_{s\in {\left\{0,1\right\}}^{n}}f\left(s\right)=\mathrm{Tr}\left(M\left({\bar{\Sigma }}^{-1}+\sigma^{-2}\sum_{i=0}^{n}s_{i}v_{i}v_{i}^{T}\right)\right),\;s.t.\;{∥s∥}_{1}\leq k, \end{array}$$

since the original problem is neither differentiable nor convex, we relax this problem to a convex optimization problem [23]

(14) $$\begin{array}{c} d^{★}=\arg\min_{d\in {\left[0,1\right]}^{n}}f\left(d\right),\;s.t.\;{∥d∥}_{1}\leq k, \end{array}$$

then let the largest *k* entries in $d^{★}$ to be included to the sampling set.

### 5.2. Experimental Results

We first evaluate the algorithms under the K-bandlimited signal assumption. Under this assumption, the entries in $\bar{x}$ indexed by K are zero, thus x can be perfectly represented by only |K| graph Fourier bases. In the following experiments we set |K|=40 and use the first 40 graph Fourier bases to form $V_{K}$.

**Figure 2:**
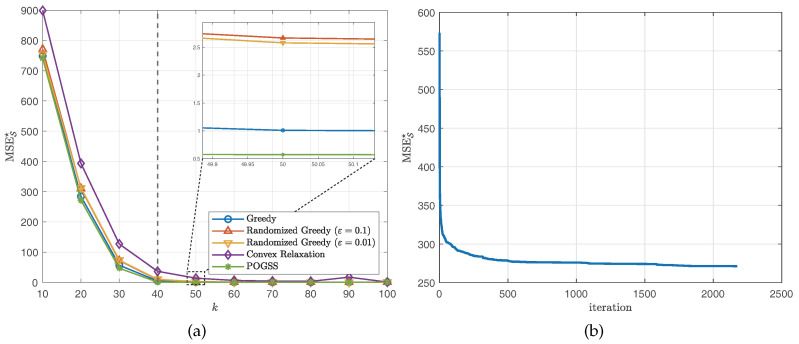
Results for bandlimited graph signals obtained on ER graphs. (**a**) ${\mathrm{MSE}}_{S}^{★}$ v.s. *k*. (**b**) Convergence curve when k=20.

**Figure 3:**
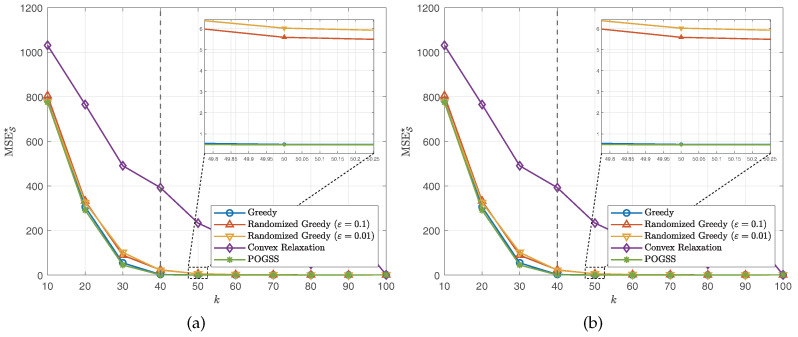
Results for bandlimited graph signals obtained on Gaussian graphs. (**a**) ${\mathrm{MSE}}_{S}^{★}$ v.s. *k*. (**b**) Convergence curve when k=20.

We set the subset size *k* from 10 to 100 with stepsize 10, then independently run the simulation 10 times and report the average optimal reconstruction MSE of the obtained subset of each algorithm. Figure 2 and Figure 3 illustrate the results obtained on ER graphs and Gaussian graphs respectively. From Figure 2a and Figure 3a we observe that POGSS and greedy-based algorithms achieve near-optimal performance, the performance convex relaxation method is worse than other method, and POGSS outperforms others for any subset size. We note that when the subset size *k* is approaching |K|=40 the MSE decreases dramatically, and when k>|K|=40 (shown in the right-hand side of the vertical dotted line) all of the methods provide the solutions with very small reconstruction MSE. Figure 2b and Figure 3b plot the convergence curve of POGSS when k=20. We can see that the MSE decreases very fast at first, and then becomes flat when k>500. However, it keeps decreasing throughout the whole process. To be precise, POGSS reaches its’ final solution at the 2144-th iteration, while $T=\lceil 2ek^{2}n\rceil =2174$.

**Figure 4:**
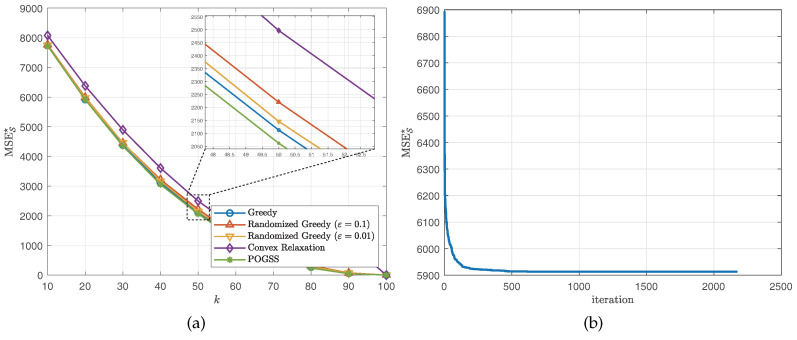
Results for non-bandlimited graph signals obtained on ER graphs. (**a**) ${\mathrm{MSE}}_{S}^{★}$ v.s. *k*. (**b**) Convergence curve when k=20.

**Figure 5:**
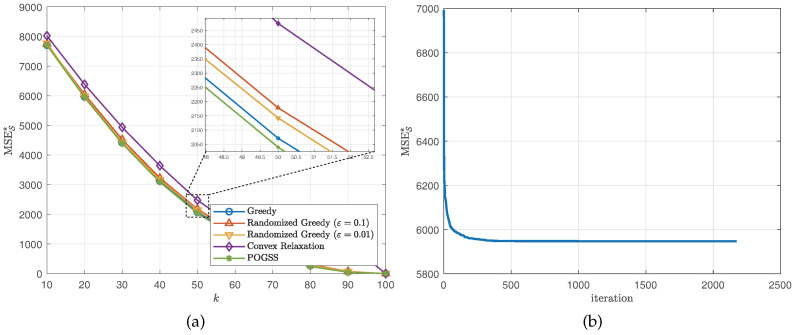
Results for non-bandlimited graph signals obtained on Gaussian graphs. (**a**) ${\mathrm{MSE}}_{S}^{★}$ v.s. *k*. (**b**) Convergence curve when k=20.

We next evaluate the algorithms for non-bandlimited graph signals, i.e., $V_{K}=V$. Similar to the bandlimited scenario, we set the subset size from 10 to 100 with step size 10 and present the average optimal reconstruction MSE of the obtained subset of each algorithm. Figure 4 and Figure 5 show the results obtained on ER graphs and Gaussian graphs respectively. From Figure 4a and Figure 5a we observe the similar results to the ones in Figure 2a and Figure 3a, where POGSS still achieves the best performance. It is worth noting that there is no “turning point” in Figure 4 and Figure 5, because the signals are non-bandlimited, and cannot be accurately reconstructed from partial observations.

**Figure 6:**
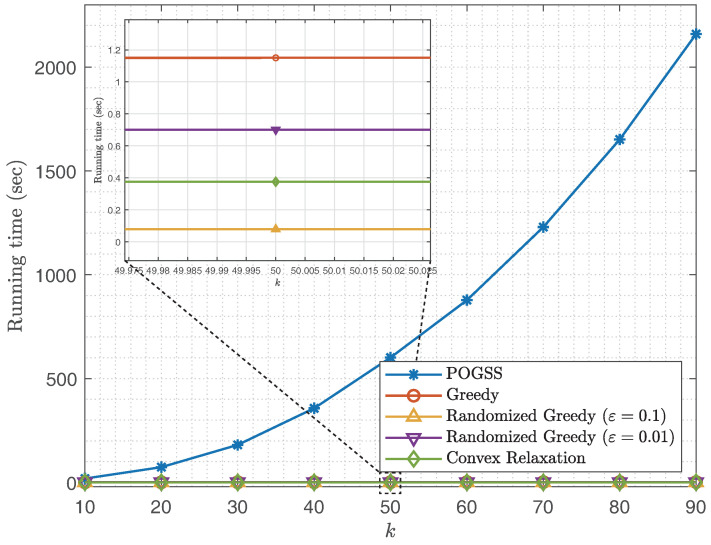
Running time v.s. *k* for non-bandlimited graph signals obtained on ER graphs.

In Figure 6 we compare the running time of different sampling set sizes. We observe that POGSS takes the most time to converge, which is as expected since Theorem 1 reveals that POGSS takse $2ek^{2}n$ iterations to converge, while greedy algorithms require only *k* iterations to do that (the time consumption of each method to finish one iteration is almost the same). Although POGSS takes the most time to find a desired solution, it is still practicable for two reasons:

1. if we fit the running time of POGSS with a quadratic function, then we have $T\approx 0.26\cdot k^{2}\;\left(\sec\right)$, thus POGSS only takes only ΔT≈5.15·k+25.77(sec) when we add 10 nodes to the current sampling set, we think it is worthwhile to find a better solution with such a time consumption.
2. In real world applications of graph signal sampling, e.g., [8], the sampling procedure is offline and the reconstruction procedure is online. We only need to sample the graph once then the entire graph signal can be always reconstructed with a small error, while the reconstruction time depends only on the size of the sampling set, rather than the sampling method.

**Figure 7:**
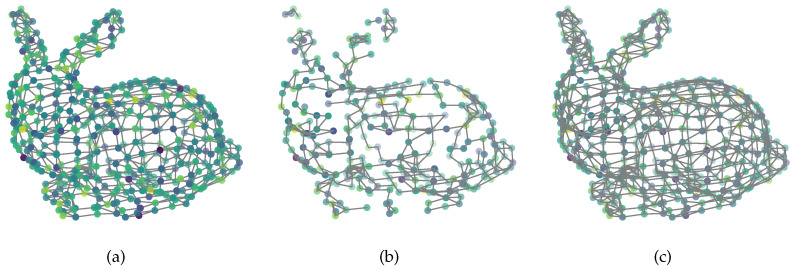
(**a**) Original noisy signal; (**b**) sampled noisy signal; (**c**) reconstructed signal.

We also provide a visual demonstration for our method. We use the stanford bunny data [6] to obtain the graph. The nodes are scanned from a 3D model, and the edges are obtained by connecting each node with its’ four nearest neighbors. The smooth graph signal is first sampled from a Gaussian distribution, and then filtered by a heat diffusion low-pass filter [24]. The resulting graph Fourier coefficients after filtering are

$$\begin{array}{c} {\hat{\lambda }}_{i}=h\left(\lambda_{i}\right)=\exp\left(-t\lambda_{i}\right), \end{array}$$

where *t* is the scaling parameter and is set to 10 in our experiment. As displayed in Figure 7a is the original smooth graph signal with Gaussian noise, the noise power is $\sigma^{2}=0.01$. There are n=453 nodes in the original graph, and we sample a subset with k=300 nodes by using POGSS to obtain Figure 7b, note that all of the edges are from the original graph. Figure 7c illustrates the optimally recovered graph signal by using (3), and the corresponding reconstruction error is ${\mathrm{MSE}}_{S}^{★}=137.2$.

## 6. Conclusions

In this paper, we consider the graph signal sampling problem, which can be formulated as a subset selection problem. The evolutionary Pareto optimization for graph signal sampling (POGSS) is proposed for solving this problem. An efficient procedure is also proposed to accelerate the evaluation of the objective function. Theoretical analysis shows that POGSS, in polynomial time, achieves the best approximation upper bound of $\left(1-e^{-\gamma_{\min}}\right)\cdot OPT+e^{-\gamma_{\min}}f\left(0\right)$ as the state-of-the-art greedy algorithm does, and an lower bound of $\gamma_{\min}$ is built as well. Simulation results demonstrate that POGSS outperforms the previous greedy algorithms.

## Data Availability

No new data were created or analyzed in this study. Data sharing is not applicable to this article.
